# Government-mandated warnings on cannabis legally sold for recreational use

**DOI:** 10.1186/s42238-020-00029-x

**Published:** 2020-07-29

**Authors:** John M. Malouff, Ben P. Schutte-Malouff

**Affiliations:** 1 University of New England School of Psychology, Armidale, NSW 2351 Australia; 2 University of Sydney, Sydney, Australia

**Keywords:** Cannabis, Health, Labeling, Marijuana, Recreational, Risks, Warnings

## Abstract

**Background:**

Frequent cannabis use can pose risks to health and safety. Multiple governments have legalized the sale of cannabis for recreational use and mandated health and safety warnings for recreational cannabis packages or signs at sales locations. The purposes of this study were to identify common themes across warnings and to compare the actual warnings with those previously recommended by cannabis experts and cannabis users.

**Methods:**

We searched Google and Google Scholar for online lists of governments that allow or will soon allow the sale of cannabis for recreational use. Using the online lists we found, we searched for laws mandating the warnings, using the search terms “mandated warnings for recreational use marijuana” in addition to the name of the jurisdiction under review. We evaluated the content of the warnings and compared them with warnings recommended by cannabis experts and by users of recreational cannabis.

**Results:**

Each search led to millions of results. Within the top results of each of the searches there were website links to official legislative websites, databases and documents of the jurisdiction under review. We used these official documents. The search revealed that 11 U.S. states and two countries allow the recreational use of cannabis and that 10 U.S. states and Canada mandate warnings on legally sold recreational cannabis. The mandated warnings can be categorized as focusing on one of nine risks: (1) negative health effects on the user, (2) harm to children or fetuses, (3) risks related to driving or operating machinery, (4) risks of habit formation leading to over-use, (5) risks relating to over-use on a single occasion, especially with regard to edible cannabis, (6) developmental risks for young people, (7) harm caused by secondary smoke, (8) risks of effects lasting several hours, and (9) risks specific to using cannabis topicals. The warnings include no graphic images and no phone number to call for help quitting.

**Conclusions:**

The warnings, as a group, parallel most warnings recommended by cannabis experts and a sample of recreational users of cannabis. The effects of the warnings are unknown, but prior research findings on warnings for cannabis and for other substances suggest potential for positive effects in raising awareness of risks and decreasing the risks. The warnings could be used in public health campaigns. Public health professionals may find it possible through research to help improve the warnings, either in presentation or in content. Cannabis researchers can use the list to identify additional risks suitable for inclusion in mandated warnings.

## Background

Legalization of cannabis sale for recreational use has grown rapidly in recent years, especially in the United States (Hansen 2019), despite serious health and safety risks being associated with use (Caulkins et al. 2016; Fischer et al. 2011; National Academies of Sciences Engineering Medicine 2017; Stoecker et al. 2018). Anticipating that governments legalizing cannabis would mandate health and safety warning, researchers published a list of warnings recommended by experts (Malouff and Rooke 2013). The 13 experts were individuals who had recently published more than one recent research article on the risks of cannabis use. They worked at universities, research institutes, or for a government. Their suggested warnings covered risks involving long-term and short-term harm to physical health and functioning, harm to mental health, danger in driving and using machinery, potential for becoming dependent, and adverse developmental effects.

Later, researchers conducted a survey in Australia in which they asked 288 adult users of cannabis to recommend government-mandated warnings. Use was illegal in Australia at the time. The users varied widely in their level of use, with some having used recreational cannabis only once and some using it most days of the week. They suggested warnings that included similar risks to those suggested by experts and also suggested warnings about risks of damage to fetuses; additionally, users suggested a mandated statement encouraging responsible use (Malouff et al. 2016). It is not known what the users meant by *responsible use*, but they might have meant something similar to what cannabis researchers have meant when using the expression: Moderation of frequency and quantity of use, use in appropriate settings, and use with respect for non-users (Lau et al. 2015).

More recently it has become apparent that some individuals experience problems from over-consuming edible cannabis because they do not expect the desired effects to have a delayed onset (Rehm 2019). Another recent development involves children gaining access at home to legally sold edible cannabis, consuming it, and suffering an adverse reaction (Dowd 2018).

The specific verbal content of warnings may play a role in how effective they are. In addition to mandating specific warnings, governments could mandate plain packaging (Goodman et al. 2019). Governments do sometimes require sellers to provide information about the THC content of cannabis, but consumers may not understand numerical presentation of the information (Hammond 2019).

One aim of the present study was to identify common themes across warnings. Another aim was to compare the actual warnings with those previously recommended by cannabis-research experts and recreational cannabis users.

## Methods

We searched Google and Google Scholar on 28 August 2019 for online lists of governments that allow or will soon allow the sale of cannabis for recreational use. Using the online lists we found, we proceeded to search for laws mandating the warnings, using only the search terms “mandated warnings for recreational use marijuana” and the name of the jurisdiction under review, e.g., Canada. Each search led to millions of results. Within the top 50 results of each of the searches there were website links to official legislative websites, databases and documents of the jurisdiction under review. We used these official documents to complete the search.

## Results

We found that 11 U.S. states have passed laws legalizing the recreational use of cannabis: Alaska, California, Colorado, Illinois, Maine, Massachusetts, Michigan, Nevada, Oregon, Washington and Vermont. Commercial sales of recreational cannabis are not allowed in Vermont (i.e., users must grow their own) and therefore no labelling requirements have been mandated. We also found that two countries, Canada and Uruguay, have legalized recreational use of cannabis at the national level.

At present, all 10 U.S. states that allow commectial sales mandate the placement of specific warnings on the products or in sales areas. Canada also mandates warnings on cannabis legally sold for recreational use. We were unable to find any mandated warnings in Uruguay. Below, we describe both the content and the format of those warnings.

We sorted the warnings into nine logical content categories upon which we both agreed. We did not use formal rules for categorizing the warnings.

The warnings focus on (1) negative health effects on the user, (2) harm to youths or fetuses, (3) risks related to driving or operating machinery, (4) risks of habit formation leading to over-use, (5) risks relating to overdose, especially with regard to edible cannabis, (6) developmental risks for young people, (7) harm caused by secondary smoke, (8) risks of effects lasting several hours, and (9) risks specific to using cannabis topicals.

Table 1 provides examples of the specific content of warnings of each type. The total number of warnings mandated by a government varied, with Canada having the most at nine. Some of the warnings refer to risks resulting from frequent or prolonged use or high THC content. The Canadian warnings contain more specific information than shown in Table 1. Table 2 provides links to the statutes and regulations that mandate the warnings.

**Table 1:** Government-mandated warnings on recreational cannabis packaging

Type of warning; states mandating	Examples covering the range of specific warnings	Government	Legal source
Negative physical and mental health effects on user; Canada, Calif, Alaska, Colo., Maine Mass, Nevada, Washington	There are health risks associated with consumption of marijuana.	Alaska	3 AAC 306
	Smoking is hazardous to your health.	Washington	WAC 314–55-105
			410 ILCS 705
	Marijuana can impair concentration, coordination, and judgment.	California	BPC Div. 10
			Ch. 12
	This product was produced without regulatory oversight for health, safety, or efficacy.	Colorado	CCR 222–2
Harm to youths or fetuses; Canada, Alaska, Calif, Colo., Mass., Mich.	Keep out of the reach of children.	Massachusetts	935 CMR 500
	WARNING: USE BY PREGNANT OR BREASTFEEDING WOMEN, OR BY WARNING: USE BY PREGNANT OR BREASTFEEDING WOMEN, OR BY WOMEN PLANNING TO BECOME PREGNANT, MAY RESULT IN FETAL INJURY, PRETERM BIRTH, LOW BIRTH WEIGHT, OR DEVELOPMENTAL PROBLEMS FOR THE CHILD.	Michigan	MRA Rule 39
	Keep away from minors.	Maine	18–691 CMR Ch. 4
Risks related to driving or operating machinery Canada, Alaska, Calif., Colo., Maine, Mass, Mich. Oregon	Do not drive a motor vehicle while under the influence of marijuana	Oregon	OLCC 845
	Do not drive or operate heavy equipment after using cannabis.	Canada	See above
	Caution must be exercised before driving or using heavy machinery	Maine	18–691- C.M.R.- ch.-1
Risks of habit formation leading to over-use; Canada, Alaska, Mass, Nevada, Washington	This product may … be habit forming.	California	See above
Risks relating to over-use in one episode; Canada, Illinois, Oregon Washinton	When eaten or swallowed, the intoxicating effects of this drug may be delayed by 2 or more hours.	Nevada	NAC Ch. 453D
	Intoxication following use may be delayed 2 or more hours	llinois	410 ILCS 705
Greater risks for adolescents and young; Canada	Adolescents and young adults are at greater risk of harm than adults	Canada	Government of Canada 2019
Secondary smoke is harmful; Canada	The smoke from cannabis is harmful.	Canada	See above
Risk of lasting effects from single use; Canada	The effects from eating or drinking cannabis … can last between 6 and 12 h	Canada	See above
Risks of cannabis topicals; Canada	Do now swallow or apply internally or to broken, irritated or itching skin	Canada	See above

**Table 2:** Sources of government-mandated warnings on packages of cannabis legally sold for recreational use

Government	Website or document name	Web address
Alaska	Regulations for the Marijuana Control Board	http://www.akleg.gov/basis/aac.asp#3.306.770
California	California Legislative Information	https://leginfo.legislature.ca.gov/faces/codes_displayText.xhtml?lawCode=BPC&division=10.&title=&part=&chapter=12.&article=
Colorado	State of Colorado: Retail Marijuana Rules	https://www.colorado.gov/pacific/sites/default/files/Amalgamated%20Retail%20Marijuana%20Rules%2001012018.pdf
Illinois	Illinois Compiled Statutes: Cannabis Regulation and Tax Act	http://www.ilga.gov/legislation/ilcs/ilcs5.asp?ActID=3992&ChapterID=35
Maine	State of Maine: Office of Marijuana Policy	https://www.maine.gov/dafs/omp/adult-use/rules-statutes/18-691-C.M.R.-ch.-1
Massachusetts	Massachusetts Cannabis Control Commission: Adult Use of Marijuana	https://www.mass.gov/doc/935-cmr-500-adult-use-of-marijuana/download
Michigan	Michigan Marijuana Regulatory Agency	https://www.michigan.gov/mra/0,9306,7-386-83994-454567--,00.html
Nevada	Nevada Administrative Code: Medical Use of Marijuana	https://www.leg.state.nv.us/NAC/NAC-453A.html
Oregon	Oregon Liquor Control Commission: Recreational Marijuana	https://secure.sos.state.or.us/oard/displayDivisionRules.action?selectedDivision=3873
Washington	Washington State Legislature: Marijuana product packaging and labeling	https://apps.leg.wa.gov/wac/default.aspx?cite=314-55-105
Canada	Government of Canada: Cannabis health warning messages	https://www.canada.ca/en/health-canada/services/drugs-medication/cannabis/laws-regulations/regulations-support-cannabis-act/health-warning-messages.html

Sources identified on 26 May 2020

^a^ See 13 ON YOUR SIDE (2020) regarding Michigan warning

Across the 11 governments that mandate warnings, the warnings cover the expert-recommended warning topics. None of the warnings suggests responsible use, as suggested by users in the study of Malouff et al. (2016).

Some governments, including California and Canada, mandate multiple warnings that must be rotated. Canada has the most comprehensive warnings, covering all nine topics. The warnings mandated by U.S. states vary in covering one to four of the types of risks. See Table 1 for information about which states mandate which types of warnings.

Only Canada has warnings about the risk of harm to mental health. No government mandates graphic warnings, which are becoming common with regards to tobacco packages (Bekalu et al. 2019), and no government mandates providing a quit-line phone number, as suggested by recreational users of cannabis in a prior study (Malouff et al. 2016).

Table 3 shows how the types of mandated warnings compared to the types of content recommended by cannabis experts and by a group of cannabis users.

**Table 3:** Mandated types of warnings compared to the types of warnings recommended by cannabis experts and types of warnings receommended by a group of cannabis users

Type of mandated warning	Number of U.S. states mandating 2020	Expert recommended 2013^a^	User recommended 2016^b^
Negative physical and mental health	6	yes	yes
Harm to youths or fetuses	5	yes	no
Risks related to driving or operating machinery	6	yes	yes
Risks of habit formation leading to overuse	4	yes	yes
Risks relating to over-use relating to single use	3	no	no
Greater risks for adolescents and youths	0	yes	yes
Secondary smoke is harmful	0	no	no
Risk of lasting effects from one use	0	no	no
Risks of cannabis topicals	0	no	no

The users also suggested recommending responsible use. Canada mandates all nine types of warnings listed in the table

^a^Malouff et al., 2013. ^b^ Malouff et al. (2016)

## Discussion

Collectively, governments that mandate warnings for legally sold recreational cannabis cover nine types of risks to users and others, and the mandated warnings meet most of the suggestions of cannabis experts and recreational users (Malouff and Rooke 2013; Malouff et al. 2016). However, individual governments vary widely in how many risks they cover in their warnings, and some U.S. states have very low coverage of the risks of use, especially compared to Canada. Tables 1 and 3 show that the majority of U.S. states do not cover most of the types of risks mentioned in warnings recommended by cannabis experts.

It is unclear why and how governments choose the warnings they mandate. It would be wise for governments to review research findings on risks and to systematically mandate warnings based on those findings. We hope that the present categorization of warnings will help encourage states and other governments to cover all relevant risks in their mandated warnings.

Researchers have just begun to examine the possible effects of health and safety warnings on legally sold cannabis. For instance, researchers found in experimental research with community samples that graphic cannabis warnings are viewed as more likely to be effective than text-only warnings and that most individuals favor including a helpline phone number in warnings (Leos-Toro et al. 2019). Other researchers found in a sample of young adults that warnings about risks to cognitive development have the highest level of perceived effectiveness (Mutti-Packer et al. 2018).

There is at present no evidence that the government-mandated warnings affect cannabis-use levels. Specific warnings may or may not have effects on individuals, including recreational cannabis users and potential users, depending on motivations for use, personality, situation of use, and other factors. The evidence that warnings on tobacco and alcohol products affect use is mixed (Noar et al. 2016; Wilkinson and Room 2009).

The currently mandated warnings could provide content for public health campaigns. Those campaigns might be especially appropriate when governments, such as in Vermont and Washington, D.C., allow growing and using cannabis for recreational use, but not sale (Government of the District of Columbia undated; Vermont General Assembly undated). As new governments legalize cannabis for recreational use, they can use the existing warnings as examples of content to include.

The warnings mandated for recreational cannabis packages or sales venues could possibly be more effective in raising risk awareness and in decreasing harmful use if they (1) cover all major risks, as indicated by the types of warnings in Table 1, with rotation of the warnings, as required by Canada, (2) include graphic warnings, which are common for mandated tobacco warnings (Bekalu et al. 2019), and (3) provide a quit-line phone number, as included in some mandated tobacco warnings (Wilson et al. 2010). Also, it might be useful to present the warnings to users using the calmer expression “health information” rather than “warnings” in order to minimize resistance to government control. See LaVoie et al. (2017) regarding the risk of reistance. These hypothesized benefits could help guide researchers in exploring the actual effects of different presentations and content.

The collected warnings provide a list of risks already covered by warnings, so that cannabis researchers can more readily identify additional risks suitable for inclusion in mandated warnings. Researchers can recommend to governments that they include these newly identified risks in mandated warnings.

Some states mandate warnings for *medical cannabis*, but we have not examined these warnings. New Mexico, for instance, recently ordered warnings regarding medical-cannabis vaping (Boyd 2019). Because the risks of use are likely similar for recreational use and medical use, mandated warnings could logically overlap to a large extent for the two types of use. However, producers of cannabis as a medicine have legal-liability reasons to voluntarily warn of risks, similar to the reasons for producers of other medical drugs (Malouff 2016). Hence, voluntary warnings might be more likely for medical cannabis than for recreational cannabis, possibly lessening the need for mandated warnings for medical cannabis.

## Conclusions

This review has shown that almost all governments that allow the sale of cannabis for recreational use mandate warnings. The mandated warnings fall into nine content categories, such as risks to fetuses. However, only some governments mandate warnings that cover a broad range of health and safety risks created by recreational cannabis use. We suggest a systematic approach for governments in choosing warnings to mandate regarding recreational cannabis. Also, we recommend further research on recreational cannabis warnings, including how best to make them effective.

## Data Availability

The authors’ electronic file of relevant laws is available from the corresponding author.
